# Turning over New Leaves

**Published:** 1889-02-16

**Authors:** 


### Turning Over New Leaves.

(Books for Review should be sent to The Editor. The Lodge, Porchester Square, W.)

### Life Without Death.*

When Mr. Besant tries to probe the future, he is not at his best; his distinctly nineteenth-century heroes and heroines seem out of place in such incongruous surroundings. A description of a time when science and socialism between them have reduced all to a dead level, is not a cheerful subject for a Christmas annual, yet this is what Mr. Besant has given us in "The Inner House." As a matter of fact this little book is both a psychical and social study, but mingled with so much that is fantastic that it is impossible to take any of it seriously, while it is not humorous enough to be accepted merely as a joke. A German professor discovers the means of prolonging life indefinitely, and certain persons possessing themselves of the discovery form a communistic colony at a new Canterbury, where they proceed to live for ever. All old people have been murdered, no births are permitted, and a population of the perennially middle-aged soon sink into a disgusting state of sloth and uncivilisation. The only thing which can rouse the least interest in the Canterbury community is the supper bell. Through chance one young girl and one man are preserved in this society, and they together work a revolution, which ends in arousing many to returning to the old life of danger and enthusiasm—enthusiasm which is founded on death. For Besant points out—as does George Eliot in "Jubal"—that it is the thouhgt of death which makes life beautiful, even as the presence of pain teaches us to appreciate pleasure. These are rather heavy tools with which to build a shilling story, and though the book is full of interest and worthy of perusal, we have read many works of Besant which we liked better.

* "The Inner House." By Walter Besant. J. W. Arrowsmith, Bristol. Price 1s.

